# Isotretinoin-Induced Thrombocytosis in a Patient With Acne Vulgaris: A Case Report

**DOI:** 10.7759/cureus.16716

**Published:** 2021-07-29

**Authors:** Afnan Alyasi, Khalid Al Hawsawi, Bashaer A Malebari, Rasha Mandili, Dhiyaa Alqasim

**Affiliations:** 1 Medicine and Surgery, Umm Al-Qura University, Makkah, SAU; 2 Department of Dermatology, King Abdulaziz Hospital, Makkah, SAU

**Keywords:** acne vulgaris, isotretinoin, thrombocytosis, platelet count, treatment, severe acne, acne treatment

## Abstract

Isotretinoin is one of the first-line medications for the treatment of acne. One of the reported side effects of isotretinoin is thrombocytopenia, in addition to other abnormalities such as incomplete blood count. However, reports on thrombocytosis associated with isotretinoin are controversial. The present report discusses the case of a patient with acne vulgaris who was treated with isotretinoin and consequently suffered from isotretinoin-induced thrombocytosis.

A 20-year-old female patient was diagnosed with acne vulgaris and started treatment with systemic isotretinoin (20 mg once daily) for one month. A baseline complete blood count was performed, as well as another blood count after one month of medication administration. Platelet count was recorded at each visit. The baseline platelet count was within the normal range; however, it was found to be elevated after one month of treatment. Accordingly, the medication was discontinued, and the platelet count returned to normal levels after one month, as measured during the monthly visit. The patient also experienced seizure episodes during treatment, which did not cease with the treatment discontinuation. Although isotretinoin-induced thrombocytosis is considered a rare side-effect for isotretinoin, it should be routinely monitored in high-risk patients and those undergoing surgeries. Further prospective studies on isotretinoin-induced thrombocytosis need to be conducted to gain a deeper insight into the various aspects related to the condition.

## Introduction

Acne vulgaris is a chronic medical condition with an etiology related to pilosebaceous inflammation [1]. The incidence of acne vulgaris is at its highest levels during adolescence, which is attributed to hormonal changes at this stage of growth [2]. Acne vulgaris is associated with multiple clinical features, most commonly erythematous pustules, and comedones [3]. Additionally, some patients may also suffer from pseudocysts or dermal nodules. Consequently, these manifestations represent a cosmetic problem for patients, leading to significant psychological stress [4].

Mild forms of acne vulgaris can be treated with topical creams or ointments. However, severe forms of the disease will require systemic treatment [5]. Isotretinoin is considered to be the first-line treatment for acne vulgaris of almost all types and severity levels [6]. It is available in both topical and systemic forms. Isotretinoin acts by reducing bacterial colonization, as well as inflammation [7]. The systemic dose of isotretinoin is calculated based on the patients’ body weight, with 0.5 mg/kg/day administered as an initial dose, and subsequently adjusted based on the clinical response and occurrence of side effects [8].

Although isotretinoin is very effective for treating acne vulgaris, it is significantly limited by the increased incidence of side effects, which is more common with systemic treatment [9]. Isotretinoin can cause gastrointestinal, retinal, and hepatological side effects, in addition to being teratogenic. Another important side effect is the abnormalities in the hematological profile [10]. Hence, it is recommended to have a baseline complete blood count and to routinely monitor any blood count changes throughout the treatment [11].

Recent studies have reported different forms of hematological abnormalities accompanying systemic administration of isotretinoin. These abnormalities include thrombocytopenia, neutropenia, and there have been rare reports of thrombocytosis as well [12]. Because of the scarcity of data on these hematological side effects, some patients could have hematological side effects that may progress into serious conditions without being observed [13].

In this report, we discuss the case of a patient who was treated with isotretinoin and suffered from thrombocytosis. This study sheds light on the importance of routine monitoring of hematological side effects of isotretinoin, particularly with systemic treatment.

## Case presentation

A 20-year-old Saudi female was diagnosed with severe acne vulgaris at our dermatology clinic in December 2019. On her routine clinic visit in October 2020, the decision was made to start treatment with systemic isotretinoin (Roaccutane®) with a dose of 20 mg once daily for one month. A baseline platelet count was requested before starting the medication, the results of which are shown in Table 1.

**Table 1:** Results of platelet count at baseline

Baseline platelet count	Reference range
392,000/μL	150,000-400,000/μL

After one month (November 2020), the patient returned to the clinic for her routine visit. A new complete blood count was requested to identify any hematological abnormalities induced by isotretinoin. The laboratory results revealed thrombocytosis, with no other abnormalities detected in the complete blood count, as shown in Table 2. A reticulocyte count and a peripheral blood smear were not done.

Accordingly, the medication was discontinued.

**Table 2:** Results of platelet count after one month of treatment

Platelet count after one month of treatment	Reference range
527,000/μL	150,000-400,000/μL

The patient also reported other non-hematological side effects. During treatment, she had recurrent episodes of seizures as diagnosed by a neurologist, who confirmed the diagnosis through an electroencephalogram (EEG); however, she did not start any anti-epileptic medication even though she had a positive family history of epilepsy.

A thorough medical history was obtained from the patient after the occurrence of these side effects. She denied any history of malignancy, chronic diseases, or any drug ingestion, other than isotretinoin, that may lead to thrombocytosis. She was not feverish during the physical examination, and we did not observe leukocytosis or any signs of infection. Also, she did not have any known drug allergies. The dermatological examination demonstrated mild nodulocystic acne in her forehead and both cheeks.

One month after the discontinuation of isotretinoin (December 2020), the patient came for her routine clinic visit. A new complete blood count was requested. The results showed that the platelet count had returned to the normal range, as shown in Table 3. However, the seizure episodes continued to occur even after one month of isotretinoin discontinuation. The patient was referred to a neurologist to initiate anti-epileptic therapy.

**Table 3:** Results of platelet count after one month of treatment discontinuation

Platelet count after one month of treatment discontinuation	Reference range
369,000/μL	150,000-400,000/μL

## Discussion

Acne vulgaris is a common dermatological condition that varies in severity among adolescents and young adults [14]. Its severity can range from mild to severe form that leads to significant psychological distress in patients. Isotretinoin is a first-line treatment for acne vulgaris, which is commonly used in its topical form, while the systemic form is used for severe cases [15]. Despite the high effectiveness of isotretinoin, it has multiple side effects, some of which can be serious. Hence, patients on isotretinoin should be routinely monitored, particularly those on prolonged or systemic therapy [16].

The present case report sheds light on the importance of routine monitoring and follow-ups with complete blood count during a systemic course of isotretinoin treatment. Our report demonstrated that thrombocytosis could occur particularly with systemic administration of isotretinoin in severe acne vulgaris patients. Other blood count abnormalities were not identified in our patient, despite those being described in the medical literature.

There is a scarcity of information on the incidence and description of isotretinoin-induced thrombocytosis. Gencoglan et al. [17] evaluated the hematological abnormalities occurring with systemic isotretinoin treatment in patients with acne vulgaris. They conducted a prospective study involving 118 patients with moderate to severe acne vulgaris. Their findings demonstrated that thrombocytosis occurred after one month of treatment while the platelet count returned to baseline value after the second month of treatment.

Similar to the findings of Gencoglan et al. [17], our patient also had thrombocytosis after one month of treatment with isotretinoin. Yet, unlike their findings, the platelet count only returned to baseline after one month of therapy discontinuation, rather than after completing the course of treatment as reported by Gencoglan et al. [17].

Moreover, Yaldız et al. [18] examined the hematological and inflammatory changes associated with the treatment of acne vulgaris with systemic isotretinoin. Their study involving 352 patients demonstrated a significant reduction in inflammatory markers in terms of leukocytes and neutrophils; however, there was a significant increase in platelet count during the treatment course. Consequently, the authors recommended continuous monitoring of blood count throughout the treatment course.

Our patient also showed a significant increase in the platelet count after one month of treatment, with no other hematological abnormalities detected. Additionally, the patient had some epileptic episodes, which were not resolved even after drug discontinuation. However, the etiology of these epileptic episodes needs further investigation to prove its correlation to isotretinoin.

Ataseven et al. [19] have presented a similar case involving a 19-year-old female patient, who developed isotretinoin-induced thrombocytosis after taking a systemic dose of 0.5 mg/kg/day, which was resolved by the discontinuation of isotretinoin. This case reported by Ataseven et al. [19] and the present case report strongly endorse the importance of routine monitoring of the complete blood count of patients. However, due to the limited nature of case reports in general, further robust studies are needed to better understand the pathophysiology of isotretinoin-induced thrombocytosis and its outcomes.

## Conclusions

Isotretinoin is a cornerstone in the treatment strategy for acne vulgaris; however, it requires a long treatment course, which increases the incidence of adverse events. Accordingly, dermatologists should regularly monitor patients on isotretinoin, particularly the complete blood count, to avoid any hematological complications. Further large studies with robust study designs are crucial to identify and gain more insight into the incidence rate and prognosis of isotretinoin-induced thrombocytosis.
